# Multi-Frame Super-Resolution of Gaofen-4 Remote Sensing Images

**DOI:** 10.3390/s17092142

**Published:** 2017-09-18

**Authors:** Jieping Xu, Yonghui Liang, Jin Liu, Zongfu Huang

**Affiliations:** National University of Defense Technology, College of Opto-Electronic Science and Engineering, Deya Road 109, Changsha 410073, China; yonghuiliang@sina.com (Y.L.); liujin1344@sina.com (J.L.); hzf_huang@126.com (Z.H.)

**Keywords:** super-resolution, blind deconvolution, remote sensing, staring imaging, MAP

## Abstract

Gaofen-4 is China’s first geosynchronous orbit high-definition optical imaging satellite with extremely high temporal resolution. The features of staring imaging and high temporal resolution enable the super-resolution of multiple images of the same scene. In this paper, we propose a super-resolution (SR) technique to reconstruct a higher-resolution image from multiple low-resolution (LR) satellite images. The method first performs image registration in both the spatial and range domains. Then the point spread function (PSF) of LR images is parameterized by a Gaussian function and estimated by a blind deconvolution algorithm based on the maximum a posteriori (MAP). Finally, the high-resolution (HR) image is reconstructed by a MAP-based SR algorithm. The MAP cost function includes a data fidelity term and a regularized term. The data fidelity term is in the L_2_ norm, and the regularized term employs the Huber-Markov prior which can reduce the noise and artifacts while preserving the image edges. Experiments with real Gaofen-4 images show that the reconstructed images are sharper and contain more details than Google Earth ones.

## 1. Introduction

Images with higher resolution are required in most electronic imaging applications, such as remote sensing, medical diagnostics, and video surveillance. During the past decades, considerable advances have been realized in imaging systems. However, the quality of images is still limited by the cost and the manufacturing technology [1]. Super-resolution (SR) is a promising digital image processing technique to construct high-resolution (HR) images from several blurred low-resolution (LR) images.

The basic idea of SR is that the LR images of the same scene contain different information because of relative sub-pixel shifts, thus, an HR image with higher spatial information can be reconstructed by image fusion. Sub-pixel motion can occur due to movement of local objects or vibrating of the imaging system, or even controlled micro-scanning [2,3]. Numerous SR algorithms have been proposed since the concept of SR was introduced by Tsai and Huang [4] in the frequency domain. However, most of researchers nowadays address the problem mainly in the spatial domain, because it is more flexible to model all kinds of image degradations [5]. Ur and Gross [6] proposed a non-uniform interpolation of multiple spatially-shifted LR images based on the generalized multichannel sampling theorem, followed by a deblurring process. Stark et al. [7] were the first to suggest an SR algorithm based on the projection onto convex sets approach (POCS). Patti et al. [8] extended the method to handle arbitrary sampling lattices and non-zero aperture time. The advantage of the POCS method is that it allows a convenient inclusion of prior knowledge into the reconstruction process. Irani and Peleg [9] estimated the HR image by back projecting the error between re-projected LR images and the observed LR images. For the ill-posed nature of the SR problem, regularization methods are widely used in SR reconstruction. Schultz and Stevenson [10] reconstructed a single HR video frame from a short image sequence using the maximum a posteriori (MAP) approach with a discontinuity-preserving image prior. They used a block-matching method to estimate the sub-pixel displacement vectors between LR images. A MAP framework for jointly estimating image registration parameters and the HR image was presented by Hardie et al. [11]. The sub-pixel displacement vectors were iteratively updated along with the HR image in a cyclic coordinate-descent optimization procedure. Zomet et al. [12] proposed a robust SR algorithm in which a median estimator was used as the gradient of the L_2_ norm cost function. Farsiu et al. [13] used a cost function in the L_1_ norm rather than modified the gradient directly to further increase the robustness.

For remote sensing applications, researchers mainly focused on single-frame SR based on learning or sparse representation for lack of multiple images of the same scene [14,15,16,17]. Nevertheless, some researchers have made their contributions. Merino and Núñez [18] proposed a method named Super-Resolution Variable-Pixel Linear Reconstruction (SRVPLR) to reconstruct an HR image from multiple LR images acquired over a long period. This algorithm was derived from drizzle [19] which was designed to work with astronomical dithered under-sampled images. A prior histogram matching on LR images and high geometrical corrections were introduced to make it compatible with remotely-sensed images. Shen et al. [20] proposed an SR method to MODIS remote sensing images, where image registration in the range and spatial domains were performed before image reconstruction. Li et al. [21] proposed a MAP-based SR algorithm with a universal hidden Markov tree model, and tested it with Landsat7 panchromatic images captured on different days. Fan et al. [22] proposed a POCS-based SR algorithm with a point spread function (PSF) estimated by a slant knife-edge method. The method was tested on Airborne Digital Sensor 40 (ADS40) three-line array images and the overlapped areas of two adjacent Gaofen-2 images.

This paper presents a technique that performs multi-frame super-resolution of Gaofen-4 satellite images. Gaofen-4 is a staring imaging satellite with high temporal resolution, and its high orbit limits spatial resolution, therefore, multi-frame SR is possible and essential. First, we build the observation model for the Gaofen-4 satellite, then we propose the pipeline of our MAP-based SR algorithm. Our algorithm consists of three steps: image registration, PSF estimation, and image fusion. Image registration is performed both in the range and spatial domains, and the Gaussian PSFs of LR images are estimated via a modified MAP-based blind deconvolution algorithm. Finally, The HR image is estimated by an image fusion method based on the MAP method with a Huber image prior, while the registration parameters are further refined along with the HR image.

The rest of this paper is organized as follows: Section 2 addresses the SR problem and details our algorithm; Section 3 presents the experimental results with synthetic and real data; Section 4 concludes the paper.

## 2. Materials and Methods

### 2.1. Observation Model

To analyze the SR problem comprehensively, an image observation model must be formulated first. For notational simplicity, the image in our method is represented as a lexicographically-ordered vector. Let us consider the desired HR image o of size LM×LN, where L denotes the downsampling factor in the observation model, and M and N are the number of rows and columns of the LR images, respectively. Generally, the observation model-related HR image and the LR images is given by [1]:

(1) $$y_{t}=D_{t}B_{t}M_{t}o+n_{t},\;\mathrm{for}\;t=1,2,\ldots ,T,$$

where $M_{t}$ is the subpixel motion matrix of size $L^{2}MN\times L^{2}MN$; $B_{t}$ represents the blur matrix of size $L^{2}MN\times L^{2}MN$; $D_{t}$ denotes the downsampling matrix of size $MN\times L^{2}MN$; $n_{t}$ is the lexicographically-ordered additive Gaussian noise vector with zero mean; and $y_{t}$ represents the lexicographically-ordered LR image vector of size MN×1.

The observation model represented by Equation (1) assumes an illumination invariant environment. However, this assumption may not be valid due to the variation of light conditions over time. The modified observation model is given by:

(2) $$y_{t}=\lambda_{1,t}D_{t}B_{t}M_{t}o+\lambda_{0,t}I+n_{t},\;\mathrm{for}\;t=1,2,\ldots ,T,$$

where λ1,t and λ0,t denote the contrast gain and illumination offset, respectively, and I is a unit vector of size MN×1. The motion matrix Mt could vary in time, while the downsampling matrix Dt and blur matrix Bt remain constant over time for most situations. Therefore, D and B are used instead of Dt and Bt, respectively. In the following of this section, we build the specific model for the Gaofen-4 satellite.

Remote sensing sensors can be modeled by rational polynomial coefficients (RPC) geometrically. However, for registering images taken by the Gaofen-4 staring imaging satellite, the scene can be effectively modeled as a planar region for its high orbit when the region of interest (ROI) is not too large and, hence, a simple affine camera model is sufficient to describe the image geometry. The affine transformation contains six parameters and can be written as:

(3) $$T=\left[\begin{array}{ccc} a_{1} & a_{2} & a_{0}\\ b_{1} & b_{2} & b_{0}\\ 0 & 0 & 1 \end{array}\right],$$

where $a_{0}$ and $b_{0}$ denote translation along x and y directions respectively, and $a_{1}$, $a_{2}$, $b_{1}$ and $b_{2}$ are the parameters representing rotation, scale and deformation. Using matrix notation, the entire affine motion parameters are represented by $\theta ={\left[a_{0},a_{1},a_{2},b_{0},b_{1},b_{2}\right]}^{T}$.

We assume a shift-invariant, convolutional blur, which can be described by the PSF in the spatial domain or the modulation transfer function (MTF) in the frequency domain. The remote sensing images can be blurred by the atmosphere, the optical system, relative motion, and pixel integration. The overall PSF is the successive convolution of all of the individual terms:

(4) $$\mathrm{PSF}={\mathrm{PSF}}_{\mathrm{atm}}⊗{\mathrm{PSF}}_{\mathrm{opt}}⊗{\mathrm{PSF}}_{\mathrm{mot}}⊗{\mathrm{PSF}}_{\mathrm{sen}},$$

which respectively summarize the atmospheric, optical system, motion and sensor contributions.

The atmosphere contributes through turbulence, light scattering, and attenuation. The spatial resolution of Gaofen-4 in the visible and near-infrared band is 50 m, and the contribution of turbulence at this spatial resolution is insignificant. The scattering and absorption of energy by the aerosols affects all spatial frequencies, therefore, causing edges in the image to be blurred. The aerosol MTF can be approximated by a Gaussian form $\exp\left[-k{\left(r/r_{c}\right)}^{2}\right]$ for r below the cut-off frequency $r_{c}$ [23].

A limited aperture size of the optical system causes a diffraction blur, and the corresponding PSF is given by the Airy disk. Other optical aberrations, such as defocus and spherical aberrations, also contribute to the optical system, which can be seen as perturbations to the Airy disk. A general optical system can be expressed as a Gaussian function:

(5) $${\mathrm{PSF}}_{\mathrm{opt}}(x,y)=\frac{1}{2\pi ab}\exp\left(-\frac{x^{2}}{2a^{2}}\right)exp\left(-\frac{y^{2}}{2b^{2}}\right),$$

where a and b denote the deviations of the two-dimensional Gaussian function along the x and y direction, respectively.

The motion-related blur results from shift and vibrations. For pushbroom and whiskbroom sensors, the PSF can be characterized by a rectangular function along the scanning direction. We assume the motion blur of the Gaofen-4 satellite can be ignored for it is a staring imager equipped with focal-plane arrays.

The finite size of the sensor pixels results in sensor blur which presents the integration of irradiance over pixels. The PSF is given by:

(6) $${\mathrm{PSF}}_{\det}(x,y)=\mathrm{rect}(x/w)\mathrm{rect}(y/w),$$

where w is the size of a pixel.

All PSF terms can are gathered into a single Gaussian term, which is given by:

(7) $$\mathrm{PSF}(x,y)=\frac{1}{2\pi \sigma^{2}}\exp\left[-\frac{x^{2}+y^{2}}{2\sigma^{2}}\right],$$

where we further assume the deviations along the x and y direction are equal and denoted by σ.

### 2.2. Image Registration

The success of the SR technique relies on the subpixel motion between images, and the image registration accuracy affects the quality of the reconstructed HR image significantly. Geometric registration and photometric registration are interactive. Most photometric registration approaches require accurate geometric registration, and geometric registration may fail when images are not aligned photometrically. We first perform geometric registration coarsely using speeded up robust features (SURF) [24], which is not invariant to image contrast gain and illumination bias. Then photometric and geometric registration are jointly performed using alternative minimization.

The relationship between the target image $f_{T}(x,y)$ and the reference image $f_{R}(x,y)$ is given by the following model:

(8) $$f_{R}(x)=P\left(G\left(f_{T}(x;\theta )\right);\lambda \right)+n(x),$$

where x denotes the spatial coordinates; θ and λ are geometric and photometric parameters in matrix form, respectively; G and P are geometric and photometric models, respectively; and n(x) is the additive Gaussian noise. In this paper, we focus on the linear photometric model and affine motion. Thus, Equation (8) becomes:

(9) $$f_{R}(x,y)=\lambda_{1}f_{T}(a_{1}x+a_{2}y+a_{0},b_{1}x+b_{2}y+b_{0})+\lambda_{0}+n(x,y),$$

where $\lambda_{1}$ and $\lambda_{0}$ denote the contrast gain and illumination bias, respectively. The goal of the image registration is to estimate these parameters via minimization of an objective function in the least square sense:

(10) $$\left\{\hat{\theta },\hat{\lambda }\right\}=\mathrm{argmin}F(\theta ,\lambda )=\mathrm{argmin}{\left\|f_{R}(x,y)-g(x,y)\right\|}_{(x,y)\in \Omega }^{2},$$

where g(x,y) is the transformation of the target image which is expressed as:

(11) $$g(x,y)=\lambda_{1}f_{T}(a_{1}x+a_{2}y+a_{0},b_{1}x+b_{2}y+b_{0})+\lambda_{0},$$

and Ω represents the region of inliers. To detect inliers, we use a geometric criterion and a photometric criterion. Geometric criterion requires that the pixels of the warped image should have the same geometric field with the reference image, i.e.,

(12) $$-N/2+1\leq a_{1}x+a_{2}y+a_{0}\leq N/2,$$

(13) $$-M/2+1\leq b_{1}x+b_{2}y+b_{0}\leq M/2,$$

where M and N are the number of rows and columns of the reference image, respectively. The photometric criterion requires that the intensity difference between the reference image and the registered target image should be small enough, that is to say:

(14) $$\left|f_{R}(x,y)-g(x,y)\right|\leq d,$$

where d is the intensity threshold and is set to 8.

The solution of Equation (10) can be obtained by an optimization algorithm, such as the conjugate gradient descent algorithm or the Gaussian-Newton method. However, it is easy to fall into a local optimal solution when the misalignment between two images is large. Thus, SURF is employed to estimate the geometric parameters coarsely, then an iterative updating strategy is used to estimate the photometric and geometric parameters more precisely. Specifically, at each iteration, the affine motion parameters are firstly updated by a new variation of the Marquardt-Levenberg algorithm, which was proposed by Thévenaz et al. [25], given the photometric parameters are fixed, then the affine motion parameters are kept fixed, and the photometric parameters are updated by the conjugate gradient method. The gradients of F(θ,λ) with respect to $\lambda_{1}$ and $\lambda_{0}$ are given by:

(15) $$\frac{\partial F(\theta ,\lambda )}{\partial \lambda_{1}}=\sum_{(x,y)\in \Omega }-2\left[f_{R}(x,y)-\lambda_{1}f_{W}(x,y)-\lambda_{0}\right]f_{W}(x,y),$$

(16) $$\frac{\partial F(\theta ,\lambda )}{\partial \lambda_{0}}=\sum_{(x,y)\in \Omega }-2\left[f_{R}(x,y)-\lambda_{1}f_{W}(x,y)-\lambda_{0}\right],$$

where $f_{W}(x,y)$ is the target image after geometric registration, but without photometric registration.

The image registration algorithm flow is shown in Algorithm 1. In the feature-matching step, the random sample consensus (RANSAC) method [26] is used to eliminate matching outliers. The stop criterion of this image registration algorithm is that values of the estimated parameters do not change anymore, which can be expressed by:

(17) $${\sum \left|\frac{{\hat{\theta }}_{k+1}-{\hat{\theta }}_{k}}{{\hat{\theta }}_{k}}\right|}^{2}+{\sum \left|\frac{{\hat{\lambda }}_{k+1}-{\hat{\lambda }}_{k}}{{\hat{\lambda }}_{k}}\right|}^{2}<\varepsilon ,$$

where $\sum^{\text{​}}$ means the sum of all elements; ${\hat{\theta }}_{k}$ represents the estimated geometric parameters after k times cycles, and so on. ε is the precision threshold; here, $10^{-4}$ is used.

**Algorithm 1.** Image registration algorithm.1***Input:*** LR images $\left\{y_{t}\right\}$.2***Output:*** Geometric and photometric parameters.3Select one image $y_{k}$ as the reference image, and detect its SURF features.4***for***
l=1,2,…,T(l≠k), ***do***5 Detect SURF features of the LR image $y_{l}$.6 Match features of $y_{l}$ and $y_{k}$, then estimate geometric parameters θ based on RANSAC.7 ***do***8   Estimate photometric parameters λ using conjugate gradient method for fixed geometric parameters θ.9   Estimate geometric parameters θ using the Marquardt-Levenberg method for fixed photometric parameters λ.10 ***until***
λ and θ do not change anymore.11***endfor***

### 2.3. PSF Estimation

The PSF of remote sensing images can be measured after being launched, or estimated via sharp edge prediction. Sometimes, appropriate sharp edges do not exist, so the PSF estimation method should be employed. We propose a modified MAP-based multi-frame blind deconvolution algorithm to estimate the PSF, which was originally proposed by Matson et al. [27]. The forward model used in this algorithm is:

(18) $$g_{t}(x)=o(x)*h_{t}(x)+n_{t}(x),\;\mathrm{for}\;t=1,2,\ldots ,T,$$

where $g_{t}(x)$ is the t-th measurement image; o(x) is the true object; $h_{t}(x)$ is the t-th PSF; $n_{t}(x)$ is the t-th additive Gaussian noise; and ∗ denotes convolution. The cost function to generate estimates of the PSFs is given by:

(19) $$U\left[\hat{o}(x),\{{\hat{h}}_{t}(x)\}\right]=\sum_{t}\frac{{\left\|g_{t}(x)-\hat{o}(x)*{\hat{h}}_{t}(x)\right\|}^{2}}{\sigma_{n,t}^{2}}+\gamma \hat{o}{\left(x\right)}^{2},$$

where γ is the regularized parameter and $\sigma_{n,t}^{2}$ is the image noise variance of the t-th frame. The minimization of the cost function is performed via the conjugate gradient method in a cyclic coordinate-descent optimization procedure. Readers can refer to [27] for more details of this blind deconvolution algorithm.

To make the above algorithm compatible with our application, we use the bicubic upsampled registered LR images as the measurement images. In order to save computational resources, no more than four measurement images are used. The estimated PSFs are reparameterized by Gaussian PSFs, which are given by Equation (7). The mean deviation of these PSFs is used in image fusion. We use the mean deviation because we assume there are no significant differences between the PSFs.

### 2.4. Image Fusion

Mathematically, the SR problem is an inverse problem whose objective is to estimate an HR image from multiple observed blurry LR images. The MAP estimator is a powerful tool to solve this type of problem. The MAP optimization problem can be represented by:

(20) $${\hat{x}}_{\mathrm{MAP}}=\arg\max p(\{o|\{y_{t}\})=\arg\max\sum_{t}\frac{p(y_{t}|o)p(o)}{p(y_{t})}.$$

Under the assumption of additive Gaussian noise, the likelihood term $p(y_{t}|o)$ can be expressed as:

(21) $$p(y_{t}|o)=\frac{1}{\sqrt{2\pi }\sigma_{n,t}}\exp\left[-\frac{{\left\|y_{t}-\lambda_{1,t}DBM_{t}o-\lambda_{0,t}\right\|}^{2}}{2\sigma_{n,t}^{2}}\right].$$

The term p(o) represents the probability density of the HR image, which restricts the solution space and introduces prior knowledge to the reconstruction. In particular, we employ a Huber Markov random field (HMRF) as the image prior. HMRF encourages piecewise smoothness, and can preserve image edges as well. The expression of p(o) is given by:

(22) $$p(o)=\frac{1}{Z}\exp\left[-\frac{v}{2}\rho (So,\alpha )\right],$$

where S denotes a gradient operator; v denotes the prior strength; α is a parameter of the Huber function; and Z is a normalization factor. The Huber function ρ is defined as:

(23) $$\rho (z,\alpha )=\sum_{i}\rho (z_{i},\alpha ),$$

(24) $$\rho (z_{i},\alpha )=\{\begin{array}{cc} z_{i}^{2}, & |z_{i}|<\alpha \\ 2\alpha |z_{i}|, & \mathrm{otherwise} \end{array}.$$

where $z_{i}$ is the i-th element of the vector z.

By substituting Equations (21)–(24) to Equation (20) and neglecting the constant term, the MAP estimator can be converted to a minimizing cost function, which is given by:

(25) $$J(o,\{\theta_{t}\},\{\lambda_{t}\})=\sum_{t=1}^{T}\frac{{\left\|y_{t}-\lambda_{1,t}DBM_{t}o-\lambda_{0,t}\right\|}^{2}}{\sigma_{n,t}^{2}}+v\sum_{z\in So}\rho (z,\alpha ),$$

**Figure 1:**
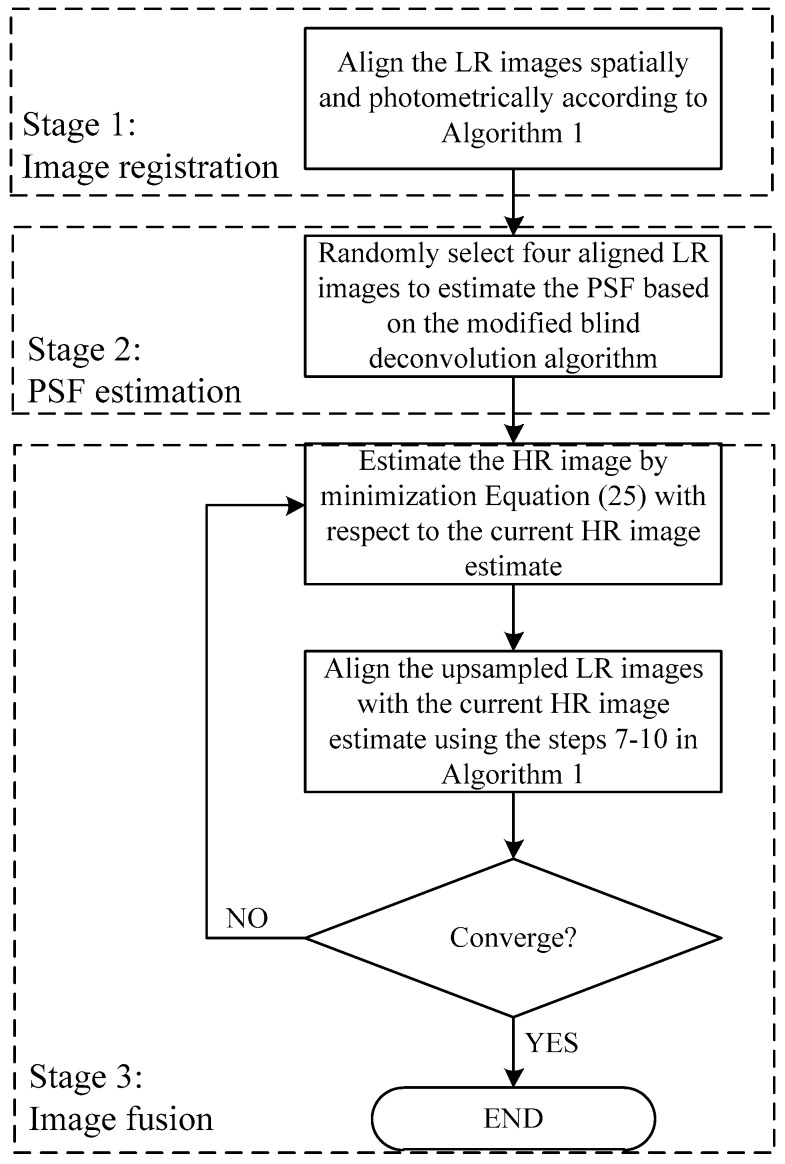
Diagram of the proposed algorithm.

The accuracy of the geometric and photometric registration is limited by the low quality of the LR images. Thus, an iterative update of the registration parameters, along with the HR image, is required. For the discrepancy of convergence rate of x, $\left\{\theta_{t}\right\}$, and $\left\{\lambda_{t}\right\}$, a cyclic coordinate-descent optimization procedure is adopted. At each iteration, the HR image is updated by minimizing Equation (25) with respect to o using a scaled conjugate gradient optimization method, given the registration parameters are fixed; then the registration parameters are updated by minimizing the cost function with respect to the registration parameters, given the HR image estimate is fixed. This minimization is completed by an image registration process, which is the same with the registration method proposed in Section 2.2. However, feature-based registration is not needed anymore, and the current HR image estimate is used as the reference image instead. The algorithm stops when the value of the function does not change. The proposed algorithm as a whole system is illustrated in Figure 1. The algorithm consists of three stages: image registration, PSF estimation and image fusion.

## 3. Results and Discussion

### 3.1. Simulation Images

We first test our algorithm using synthetic images. The quantitative evaluation of the algorithm is achieved by computing the root mean square error (RMSE) and Structural Similarity (SSIM). RMSE is defined as:

(26) $$\mathrm{RMSE}=\sqrt{\frac{1}{m}{\left\|\hat{o}-o\right\|}^{2}},$$

where o and $\hat{o}$ are the original image and the reconstructed HR image respectively, and m is the total number of pixels of the original image. The SSIM index is defined as: [28]

(27) $$SSIM(o,\hat{o})=\frac{\left(2\mu_{o}\mu_{\hat{o}}+c_{1})(2\sigma_{o\hat{o}}+c_{2}\right)}{\left(\mu_{o}^{2}+\mu_{\hat{o}}^{2}+c_{1})(\sigma_{o}^{2}+\sigma_{\hat{o}}^{2}+c_{2}\right)}$$

where $\mu_{o}$ and $\mu_{\hat{o}}$ are the means and $\sigma_{o}$ and $\sigma_{\hat{o}}$ are the standard deviations of the original and the reconstructed HR image, $\sigma_{o\hat{o}}$ is the covariance of o and $\hat{o}$, $c_{1}=60$ and $c_{2}=180$.

A sequence of LR images are generated by the following procedure:

(i) Apply random affine transformations generated by their SVD decomposition R(β)R(−ϕ)ΛR(ϕ) with translations added, where R(β) and R(ϕ) are rotation matrices with β and ϕ uniformly distributed in the range of $\left(-5^{\circ },\;5^{\circ }\right)$; Λ is a diagonal matrix with diagonal elements uniformly distributed in the range of (0.95, 1.05); and the additive horizontal and vertical displacements are uniformly distributed in the range of (–5, 5) pixels.
(ii) Blur the images with a Gaussian point spread function (PSF) with the standard deviation equal to 1.
(iii) Clip images to 0.8 times of the size both horizontally and vertically, so that only overlapped regions are left.
(iv) Downsample the images by a factor of L.
(v) Apply photometric parameters with uniformly-distributing contrast gain in the range of (0.9, 1.1) and bias in the range of (−0.1, 0.1)×max(o).
(vi) Make the SNR of the images become 30 dB by adding white Gaussian noise with zero mean.

**Figure 2:**
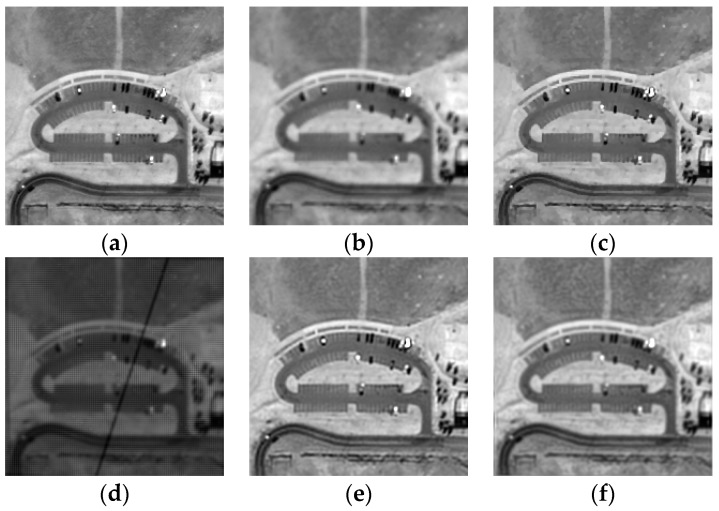
Simulation results of different methods. (**a**) Origin HR image; (**b**) the bicubic upsampled reference image; (**c**) the proposed method; (**d**) the proposed method without photometric registration; (**e**) IBP; (**f**) Farsiu’s algorithm.

A QuickBird 2 remote sensing image with a size of 256×256 is used as the origin HR image, and the downsampling factor is equal to 2. We use six LR images to reconstruct an HR image. The Huber function parameter α is set to 0.04, and the prior strength parameter v is set to 10. The estimated deviation of the total PSF is 1.12. Our method is compared with the bicubic interpolation, the iterative back projection (IBP) approach proposed by Irani and Peleg [9] and Farsiu’s robust SR algorithm [13]. Note that the original IBP and Farsiu’s algorithm do not consider the variations of the illumination in the image registration stage, thus we use our image registration algorithm instead. Figure 2 shows the original image and the reconstructed images by different methods. Compared with the bicubic upsampled image, the HR images reconstructed by all SR methods are sharper and contain more details. The reconstructed images are further compared quantitatively in RMSE and SSIM, as is listed in Table 1. The performance of our method and IBP is close, and better than the bicubic interpolation and Farsiu’s algorithm. Additionally, without photometric registration, many artifacts appear in the reconstructed HR image, as is shown in Figure 2d.

### 3.2. Real Gaofen-4 Images

**Figure 3:**
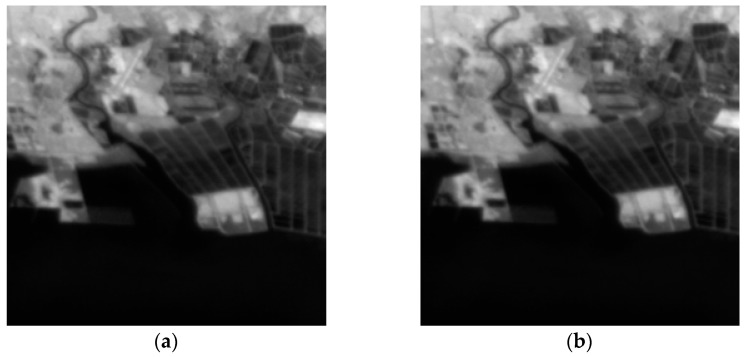
Two LR images: (**a**) captured at 11:10:20; and (**b**) captured at 11:20:21.

The second experiment is carried out with ten 5-band Gaofen-4 images without ortho-rectification. The images were all captured on 3 March 2017, from 11:10:20 to 11:20:21. The original size of full-view images is 10,240 × 10,240, and we chip the region of interest (ROI) with a size of 256 × 256. This region is an offshore aquaculture area, and the images captured at 11:10:20 and 11:20:21 are shown in Figure 3.

The image captured at 11:10:20 is used as the reference image, and the other images are aligned to it. Although the images were captured over a short period, photometric registration is needed. The criterion used to evaluate the registration accuracy is signal to noise ratio (SNR), which is defined as:

(28) $$\mathrm{SNR}=10\mathrm{lg}\frac{{\left\|f_{R}(x)\right\|}^{2}}{{\left\|f_{R}(x)-g(x)\right\|}^{2}},$$

where g(x) is the target image after registration, and $f_{R}(x)$ is the reference image. The registration results are listed in Table 2. The images are numbered according to their capture time and the first LR image is used as the reference image. All images have similar registration accuracy with both geometric and photometric registration while, without photometric registration, the SNRs are lower, and become lower and lower as time progresses.

**Figure 4:**
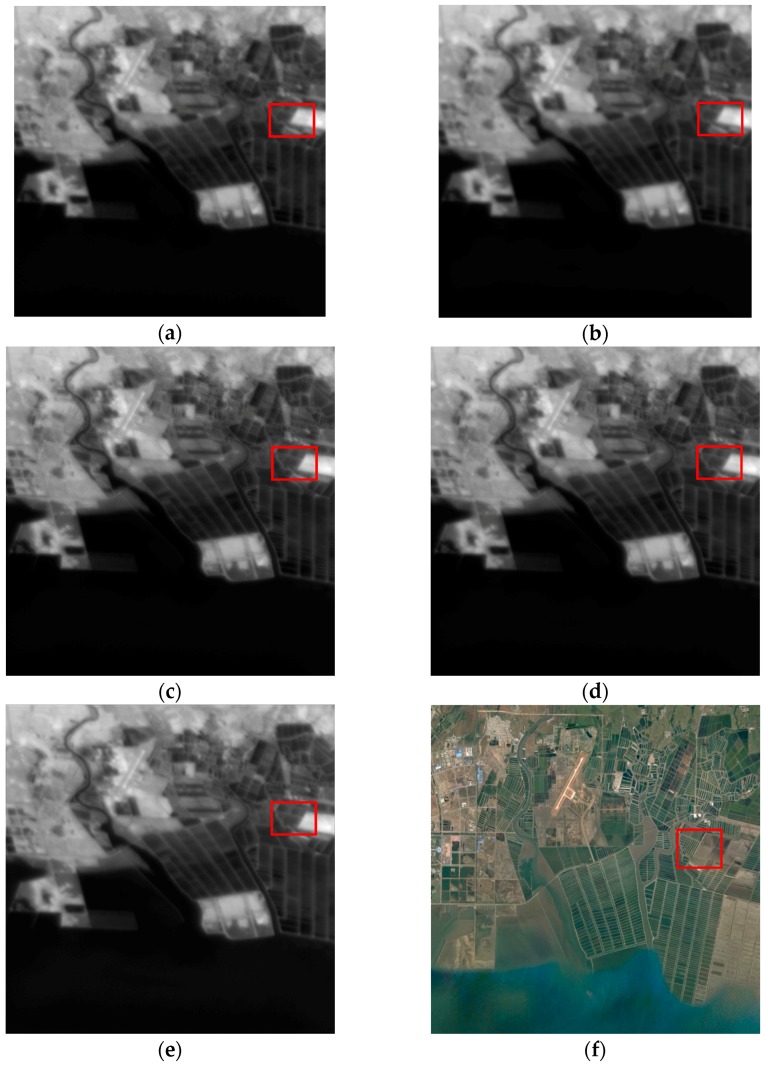
Experimental results of an offshore aquaculture area. (**a**) The bicubic upsampled reference image; (**b**) the added image after registration; (**c**) the HR image reconstructed by the proposed method; (**d**) the HR image reconstructed by IBP; (**e**) the HR image reconstructed by Farsiu’s algorithm; and (**f**) the image chipped from Google Earth.

The Huber function parameter α is set to 0.04, and the prior strength parameter v is set to 10. The estimated deviation of the total PSF is 1.21. The experimental results are shown in Figure 4.

**Figure 5:**
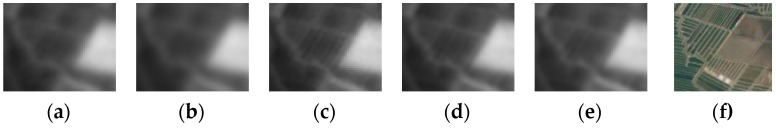
The regions denoted by the rectangles in Figure 5. (**a**) The bicubic upsampled reference image; (**b**) the added image after registration; the HR images reconstructed by (**c**) the proposed method, (**d**) IBP and (**e**) Farsiu’s algorithm; (**f**) the image chipped from Google Earth.

To validate our reconstructed HR image, we chip an image of the same zone from Google Earth, which was captured on 16 September 2015. The reconstructed HR image is clearer and contains more details which corresponds to Google Earth. To see more clearly, the regions denoted by the rectangles in Figure 4 are shown in Figure 5 without zooming. It is clear that the HR image reconstructed by our method is the sharpest.

**Figure 6:**
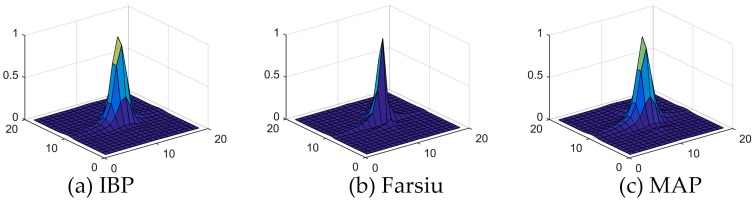
The PSFs between the upsampled reference image and the reconstructed HR images of the offshore aquaculture area.

Quantitatively evaluating the performance of the proposed algorithm is not easy in real image applications, for there are no true original images. A commonly used strategy to solve the problem is to treat the image of an inferior resolution level such as the panchromatic image as the true original image. But the strategy is not applicable for Gaofen-4 images, because the panchromatic and multi-spectral images of Gaofen-4 have the same spatial resolution. Theoretically, the reconstructed HR image should be sharper than the bicubic upsampled image. Thus we assume the upsampled image is the blurred version of the reconstructed HR image and estimate the PSF between them. The PSF estimation is completed by the PSF estimation algorithm proposed by Matson et al. which has been described in Section 2.3. Then the PSF is fitted to the Gaussian function. A larger deviation of the Gaussian function means a sharper reconstructed HR image. The estimated PSFs for IBP, Farsiu’s algorithm and our MAP-based SR algorithm are shown in Figure 6. The deviations of the fitted PSFs are 1.0, 0.9 and 1.0 respectively, which means our method has a close performance with IBP, and is better than Farsiu’s algorithm.

**Figure 7:**
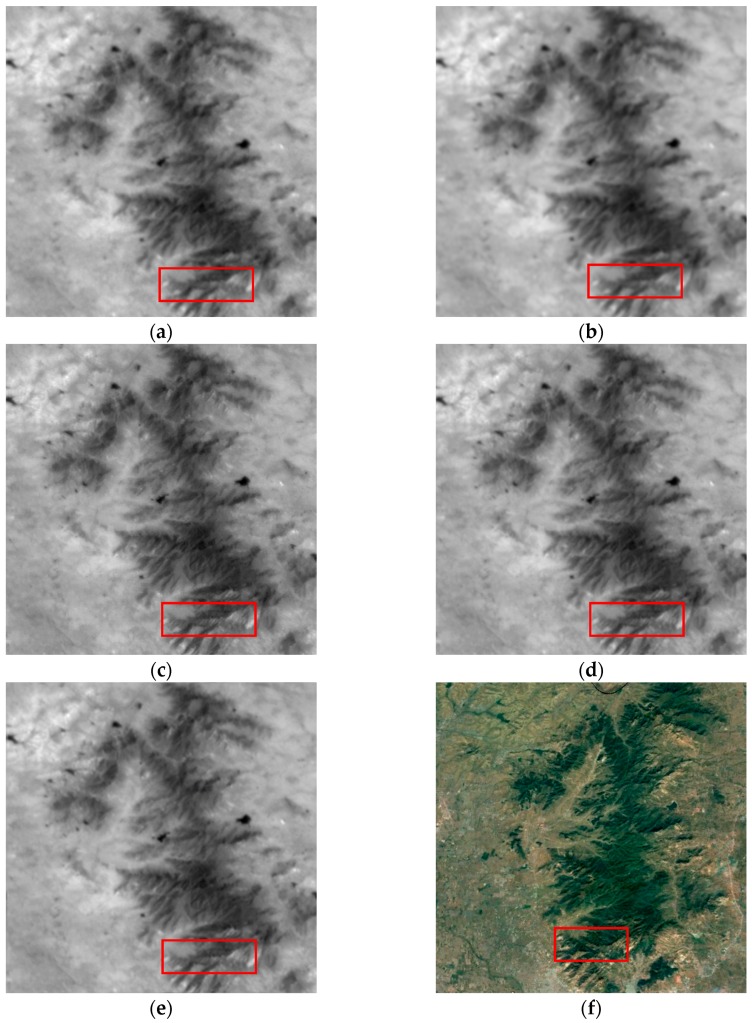
Experimental results of the hilly area. (**a**) The bicubic upsampled reference image; (**b**) the added image after registration; the HR images reconstructed by (**c**) the proposed method, (**d**) IBP and (**e**) Farsiu’s algorithm; (**f**) the image chipped from Google Earth.

**Figure 8:**
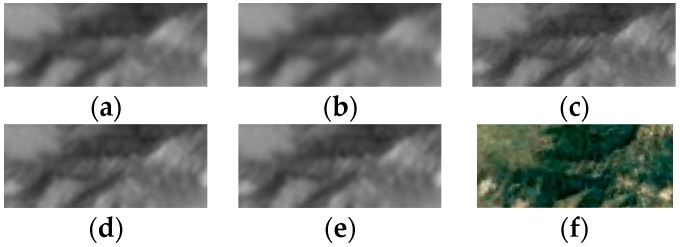
The regions denoted by the rectangles in Figure 7. (**a**) The bicubic upsampled reference image; (**b**) the added image after registration; the HR images reconstructed by (**c**) the proposed method, (**d**) IBP and (**e**) Farsiu’s algorithm; (**f**) the image chipped from Google Earth. The Google Earth image is rotated to be compared with the Gaofen-4 images without ortho-rectification.

We extract another hilly region of interest with a size of 256 × 256 to further test our algorithm. The parameters are set the same as the previous experiment. The estimated deviation of the total PSF is 1.19, which is almost same with the previous experiment. The experimental results are shown in Figure 7. The Google Earth image was captured on 20 April 2017. To see more clearly, the regions denoted by the rectangles in Figure 7 are shown in Figure 8. Compared with other methods, our method results in the sharpest edges.

**Figure 9:**
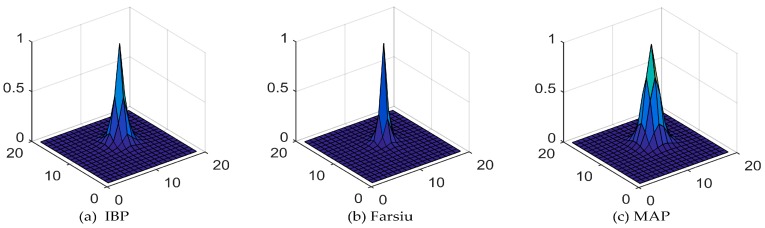
The PSFs between the upsampled reference image and the reconstructed HR images of the hilly area.

The PSFs between the HR images and the upsampled reference image of the hilly area are shown in Figure 9. The deviations of the fitted PSFs for IBP, Farsiu’s algorithm and our MAP-based SR algorithm are 0.98, 0.82 and 1.0 respectively, which means our method yields the best SR result.

### 3.3. Further Discussion

**Figure 10:**
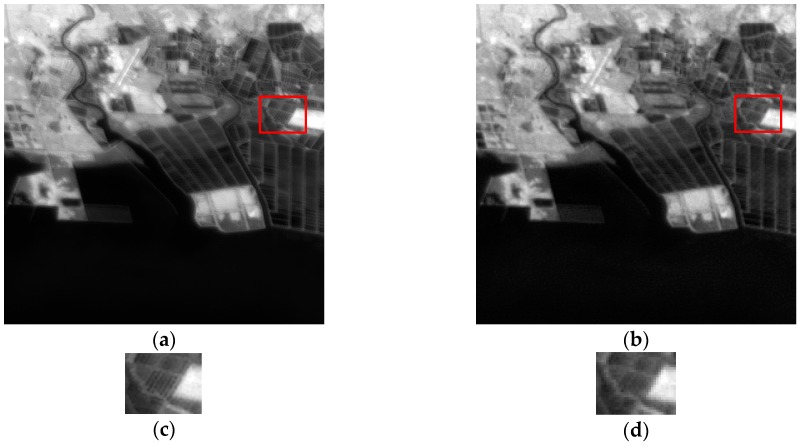
Results of image enhancement. (**a**) SR + image enhancement; (**b**) image enhancement of the reference image; (**c**) and (**d**) are the regions denoted by the rectangles in (**a**) and (**b**) respectively.

The deviation of the PSF between the HR image and the upsampled image is less than the deviation estimated in the PSF estimation stage, i.e., the blur is not removed completely. A visually better image can be generated by contrast manipulation plus sharpening after the SR reconstruction. We take the real Gaofen-4 images of the offshore aquaculture area as an example. The result of enhancing the HR image reconstructed by our SR method (Figure 4c) is shown in Figure 10a. For comparison, the reference LR image is enhanced directly without SR reconstruction, and the result is shown in Figure 10b. The regions denoted by the rectangles in Figure 10a,b are shown in Figure 10c,d. Comparing Figure 4, Figure 5 and Figure 10, we note that the enhanced LR image is visually better than the LR image and the reconstructed HR image, but contains less details. That is to say, the SR algorithm reveals more information contained in the LR images.

## 4. Conclusions

In this paper, we propose an SR algorithm to reconstruct an HR image from multiple LR images captured by the Gaofen-4 staring imaging satellite. An appropriate observation model with photometric parameters is built. The registration with photometric adjustment improves the registration accuracy and the quality of the final reconstructed HR image. A MAP-based blind deconvolution algorithm is modified to estimate the imaging PSF which is reparameterized by a Gaussian function. Finally, the PSF is used to estimate the HR image. Experimental results with synthetic and real images show that the reconstructed HR image reveals more details which cannot be seen in LR images.

## Figures and Tables

**Table 1:** The RMSE and SSIM between the original and reconstructed images obtained from bicubic interpolation, IBP, Farsiu’s method and the proposed method.

Methods	Bicubic	Our Method	IBP	Farsiu
RMSE	16.44	13.36	13.94	15.90
SSIM	0.917	0.941	0.940	0.928

**Table 2:** Registration results. The row of SNR 1 lists the registration results with both geometric and photometric registration, and the row of SNR 2 lists the registration results with only geometric registration.

Image Number	2	3	4	5	6	7	8	9	10
SNR 1	40.5	39.7	41.0	40.3	40.6	40.1	39.7	38.5	39.1
SNR 2	39.9	38.1	38.0	36.4	35.5	34.4	33.4	32.2	31.7
